# In Vitro Antibacterial Activity and Mechanism of Silver Nanoparticles against Foodborne Pathogens

**DOI:** 10.1155/2014/581890

**Published:** 2014-09-17

**Authors:** S. Rajeshkumar, C. Malarkodi

**Affiliations:** ^1^Department of Biochemistry, Adhiparasakthi College of Arts and Science, Kalavai, Vellore District 632506, Tamil Nadu, India; ^2^Environmental Nanotechnology Division, Sri Paramakalyani Centre for Environmental Sciences, Manonmaniam Sundaranar University, Alwarkurichi 627 412, Tamil Nadu, India

## Abstract

Biosynthesis of silver nanoparticles using *Planomicrobium* sp. and to explore the antibacterial activity against food borne pathogenic bacteria *Bacillus subtilis*, (3053) *Klebsiella planticola* (2727) *Klebsiella pneumoniae* (MAA) *Serratia nematodiphila* (CAA) and *Escherichia coli*. In the current studies, 1 mM of silver nitrate was added into 100 mL of *Planomicrobium* sp. culture supernatant. The bioreduction of pure AgNO_3_ was characterized by UV-visible spectroscopy, X-ray diffraction analysis (XRD), scanning electron microscopy (SEM), energy-dispersive analysis (EDS), transmission electron microscopy (TEM), and Fourier transform infrared (FT-IR) analysis. The formation of silver nanoparticles was confirmed by the presence of an absorption peak at 400 nm using UV-visible spectrophotometry. The morphology and size of the silver nanoparticles was monitored by TEM and SEM. Crystal structure was obtained by carrying out X-ray diffraction studies and it showed face centered cubic (FCC) structure. The bactericidal effect of silver nanoparticles was compared based on diameter of inhibition zone in well method. Bacterial sensitivity to nanoparticles a key factor in manufacture the suitable for long life application in food packaging and food safety. Food safety is a worldwide health goal and the food borne diseases get a main disaster on health. Therefore, controlling of bacterial pathogens in food is credit of harms associated to health and safety.

## 1. Introduction

Nanotechnology in the food manufacturing can get a number of forms. It takes in the use of nanotechnology in food packaging materials. Nanotechnology will increase antimicrobial covering for food products. The nanoparticles are dispersed during the synthesis and are capable to building block CO_2_, O_2_, and moisture from reaching fresh fruits, vegetables, meats and other foods. Packaging can give to the control of bacterial growth in food stuffs [1], which can the way to in the container of pathogenic bacteria, illness and disease. A serious problem in food packaging is that of migration and permeability [2, 3]: no stuff is totally resistant to water vapor, atmosphere level, or moisture controlled within the food being packaged. Orange juice is one of the most globally accepted fruit products. Recently several studies have been conducted to develop nonthermal processing techniques as replacement for thermal processing to keep the freshness of the juice along with extending its shelf life. Though some techniques are capable of decontaminating orange juice, recently nanotechnology introduced in the food packaging industry can potentially provide solutions to food packaging challenges. The nanocomposite LDPE films containing Ag and ZnO nanoparticles were synthesized by melt mixing in a twin-screw extruder. Orange juice was sterilized and was inoculated with 8.5 log cfu/mL of* Lactobacillus plantarum* [4]. Antibiotic resistance is one of the world's most essential product for public healthcare problems. In modern decades, almost every alternative of microbes has grown to be stronger and less exposed to antibiotic treatment, intimidating new strains of infectious diseases or superstrains that are equally more expensive to treat and more complicated to heal. Drug-resistant bacteria are raising pathogens whose resistance profiles at hand a foremost challenge for containing their spread and their collision on human health [5]. Nanotechnology has energetically developed because an important field of novel research with possible effects in biomedical technology and electronics [6]. It is implicit that the properties of silver nanoparticles are determined by their size, shape, composition, crystalline, and structure. Among the silver nanoparticles have been widely used as sensor [7], photo catalyst [8], and antibacterial agent [9]. There have been few methods such as microemulsion [10], electrospinning [11], and ultrasound radiation [12] which are available to synthesize the silver nanoparticles [13]. The metal nanoparticles are broadly used in several biomedical and bioelectrochemical applications due to their extraordinary electrocatalytic activity. It is well-known to make silver nanoparticle is an effective antibacterial agent and possesses a strong antimicrobial activity against bacteria, viruses and fungi; even however the mechanism and way of action are still not well known [14].

There are several methods programmed like food packaging, manufacturing, and agriculture, which can considerably reduce the pathogenic bacterial strains in food. However, still, the role of pathogen detection method is vital, which is the solution to the prevention of human health and safety. The present methods working for foodborne pathogen detection above the past decades to the in recent times will discuss by importance their strengths and limitation.

The current investigation aims to evaluate toxicity of well characterized silver nanoparticles against foodborne pathogens. Food spoilage is the report of bactericidal activity on the food medium causing breakdown of food components. The present reports have shown the various types of food pathogenic bacteria resistant found to be different antibiotics. The bacterial strains such as* Bacillus subtilis *(3053),* Klebsiella planticola *(2727)* Klebsiella pneumoniae* (MAA)* Serratia nematodiphila* (CAA) and* E. coli *are the major microorganisms which spoil the food. Contamination of food products by using these bacteria is able to cause health hazards to human beings.

## 2. Materials and Methods

### 2.1. Isolation and Identification of Bacteria

Ice cream samples were collected from milk market. They were serially diluted and spread on nutrient agar plates. The plates were then incubated at 27°C for 1 week. The isolated microorganism is* Planomicrobium* sp.

### 2.2. Extracellular Synthesis of Silver Nanoparticle

*Planomicrobium* sp. was grown in 100 mL nutrient broth and incubated at 35°C for 24 hrs. The overnight culture broth centrifuged at 6000 rpm for 10 minutes. The cell free supernatants were collected and 1 mM of silver nitrate was mixed and the solution was incubated at 35°C for 24 hrs. The spectrum of the sample was measured by UV-visible spectrophotometer.

### 2.3. Characterization of Silver Nanoparticle

The bioreduction of Ag ions in aqueous solution was monitored by UV-vis spectra of the solution between 300 nm and 600 nm using Perkin-Elmer spectrophotometer. The nanoparticles scanned the infrared in the region of 4000–400 cm^−1^ Fourier transform infrared spectrometer (Thermo Nicolet Model, 6700). The Ag nanoparticle suspension was air-dried on the specimen grid and was observed with a JEOL JEM-1010 Scanning Electron Microscope. The crystalline phases of the products were determined by X-ray powder diffractometer (Seifert-3000p). In the energy-dispersive X-ray analysis, the Ag nanoparticle was dried on a carbon coated copper grid and performed on a HITACHI SU6600 model.

### 2.4. Antibacterial Activity of Silver Nanoparticles

#### 2.4.1. Broth Dilution Method

The antibacterial activities of the silver nanoparticles were examined against* B. subtilis *(3053),* K. planticola* (2727),* K. pneumoniae* (MAA),* S. nematodiphila* (CAA), and* E. coli* in Luria Bertani broth (LB). The 24-hour-old bacterial cultures were inoculated into LB broth supplemented with various concentrations (250 *μ*L, 500 *μ*L, and 750 *μ*L) of silver nanoparticles. The NPs free LB broth was used as a control. The broth containing conical flasks was incubated at room temperature under stirring for 24 hr and the vulnerability of the tested organisms was observed by taking optical density at 600 nm for various time intervals.

#### 2.4.2. Well Diffusion Method

The silver nanoparticle synthesized using* Planomicrobium* sp. was tested for antimicrobial activity by agar well diffusion method against pathogenic microbes for* B. subtilis* (3053),* K. planticola* (2727),* K. pneumoniae* (MAA),* S. nematodiphila* (CAA), and* E. coli*. The pure cultures of bacteria were subcultured on nutrient broth. Each strain was swapped homogeneously onto the individual plates using sterile cotton swabs. Wells of 10 mm diameter were on Muller Hinton agar using gel puncture. Different concentration of silver nanoparticle 30 *μ*L, 60 *μ*L, and 90 *μ*L was poured on each well. After 24 hours incubation the various levels of zone of inhibition was measured. Three replicates of experiments were carried out.

## 3. Result and Discussion

### 3.1. Visual Observation

**Figure 1:**
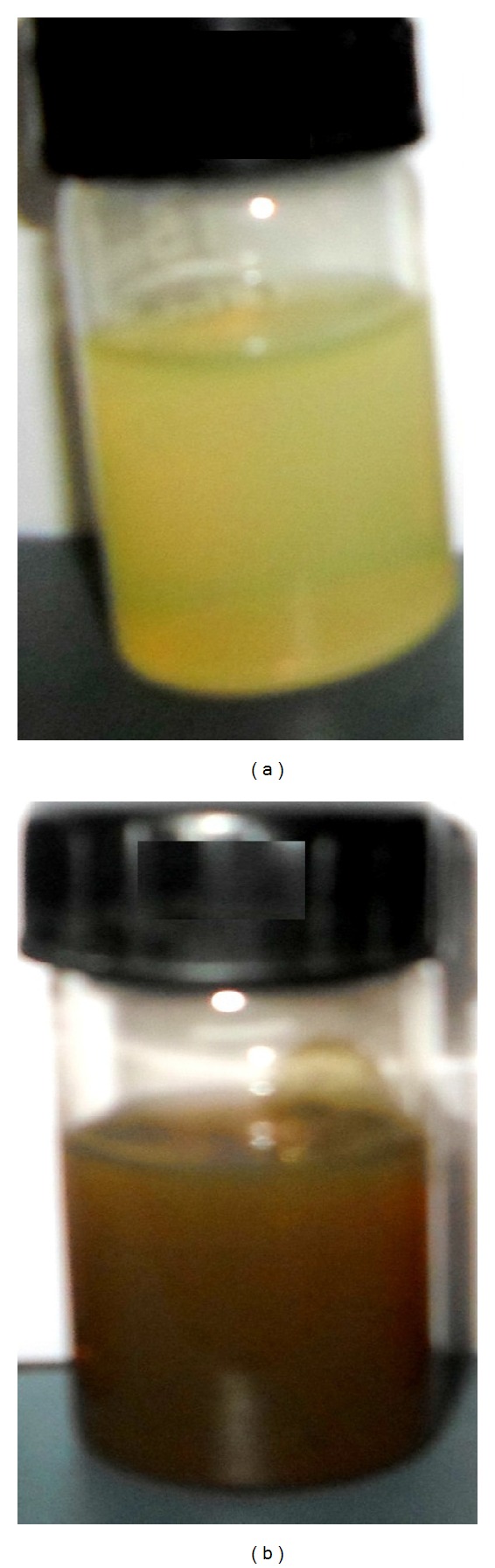
Biosynthesis of silver nanoparticles (a) and culture supernatant of* Planomicrobium* sp. (b) after the addition of AgNO_3_ at 24 hrs incubation.

The formation of silver nanoparticles was preliminarily confirmed by the change of color of the solution (Figures 1(a) and 1(b)). The color change of yellow to brown colour of the aqueous solution exhibits due to the excitation of the surface plasmon resonance. The detailed study on extracellular biosynthesis of silver nanoparticle by* Planomicrobium *sp. was carried out in this work.  Figure 1(a) shows the* Planomicrobium *sp., biomass and Figure (b) shows biomass after treatment with 1 mM silver nitrate solution. The color changes from yellow to dark brown indicating the formation of silver nanoparticles at 24 hrs of incubation. The appearance of dark brown color at 24 hrs of incubation confirms the reduction of silver nitrate into silver NPs using culture supernatant of* Planomicrobium *sp. The formation of dark brown color is the indication of synthesis of silver NPs. After 24 hrs, no charge occurred which indicates that the silver nanoparticles synthesis process was completed. After 24 hrs, the light brown color turns into dark brown color which shows the reduction of silver metal ions into AgNPs using the bacteria* Planomicrobium* sp. The color was changed into dark brown after the 24-hour-incubation due to the excitation of free electrons in the reaction mixture [15]. At 24 hrs, the stability of brown color and precipitation of particles depicted that silver nitrate completely reduced by the biomolecules. The color changes depending upon the incubation time (6–24 hrs), size, and shape of the nanoparticles.

### 3.2. UV-Vis Spectral Analysis

**Figure 2:**
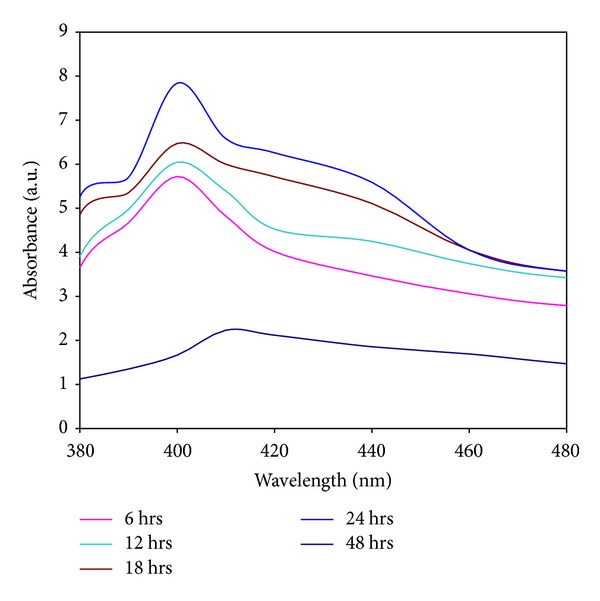
UV spectra of extracellularly synthesized silver nanoparticles using culture supernatant of* Planomicrobium *sp. show that the SPR band at 400 nm indicates nanoparticles synthesis.

The formation and stability of the reduced silver nanoparticles in the colloidal solution were monitored by using UV-vis spectral analysis. The UV-vis spectra of silver nanoparticles synthesized by using* Planomicrobium *sp. was shown in Figure 2. The silver nitrate solution was treated with* Planomicrobium *sp. at different time intervals (6, 12, 18, 24, and 48 h) assuming that different growth phase plays an important role in silver nanoparticle synthesis process. The surface plasmon resonance (SPR) band of silver occurs at 400 nm. Generally in biomass, Ag NO_3_ reduces silver nanoparticles and settles down at the bottom of the conical flask. As the size of the silver nanoparticles increases, the color of the solution varied from yellow to brown color precipitation. After 48 hrs of incubation, the rate of nanoparticles formation was reduced. After 24 hrs, the absorbance was gradually decreased which indicates that the silver nanoparticles synthesis process was completed. The silver nanoparticles are formed in the reaction mixture due to the effect of active biomolecules of microorganism and the present report was correlated with the report of* Aspergillus fumigatus* [16]. After 24 hrs, no high absorbance occurred which indicates that the reduction process of silver metal ions into silver NPs was completed. The attribute surface plasmon absorption bands are noticed at 400 nm and rising of nanoparticles size can also affect the SPR band broadening [17]. Appearance of this absorption shoulder together with hump at 420 nm indicates the presence of nanocrystallites with different sizes. This observation is well supported by TEM analysis which shows the presence of different types of particles, respectively. Similar result was reported by using the* Aspergillus flavus* [18].

### 3.3. XRD

**Figure 3:**
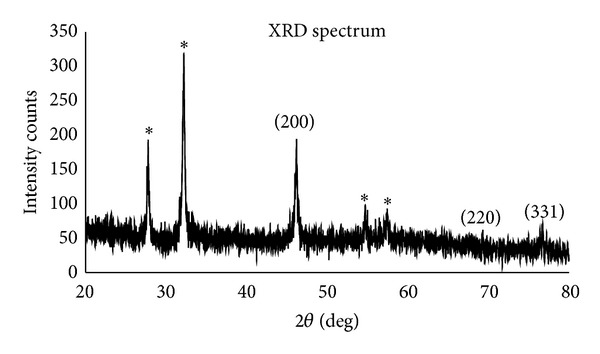
XRD pattern of extracellular synthesized silver nanoparticles using* Planomicrobium *sp.

XRD patterns obtained for the silver nanoparticle synthesized using* Planomicrobium* sp. were shown in Figure 3. There are three intense peaks in the whole spectrum of 2*θ* values ranging from 20 to 70. A comparison of our XRD spectrum with the standard confirmed that the silver nanoparticles formed in the present study in the form of nanocrystals, as evident from the peaks at 2*θ* values 44°, 64.5°, and 77° integrated intensity values of (200), (220), and (311) for cubic silver phase, respectively. These agree with those reported standards (JCPDS file number 84-0713). The four unassigned impurities peaks are observed at 28°, 32°, 54°, and 56° which may be attributed to other organic substances in culture supernatant. The XRD pattern thus clearly shows that the silver nanoparticles formed by the reduction of Ag^+^ ions by* Planomicrobium *sp. are crystalline in nature. The broadening of Bragg's peak indicates the formation of silver nanoparticles. The mean particles diameter silver nanoparticle was calculated from the XRD pattern according to the line width of the (220), refraction peak using the Scherer equation:

(1) $$\begin{array}{c} D=\frac{0.94\lambda }{\beta \left(\mathrm{Cos}\theta \right)}, \end{array}$$

where *β* is the full width at half maximum (FWHM), *λ* is the X-ray wavelength, and *θ* is the reference peak width at angle; *D* is the average crystallite area size perpendicular to the sparkly planes. It is revealed that the capping agents play an important character in affecting the crystal field bend, stability breaking, and a special role on the modification of crystal segment of silver nanoparticles during synthesis. The XRD pattern of these peaks indicates that the silver nanoparticles are crystalline in nature and some of the unassigned peaks were observed; it may be due to the fewer biomolecules of stabilizing agents such as enzymes or proteins of the bacteria [19].

### 3.4. SEM

**Figure 4:**
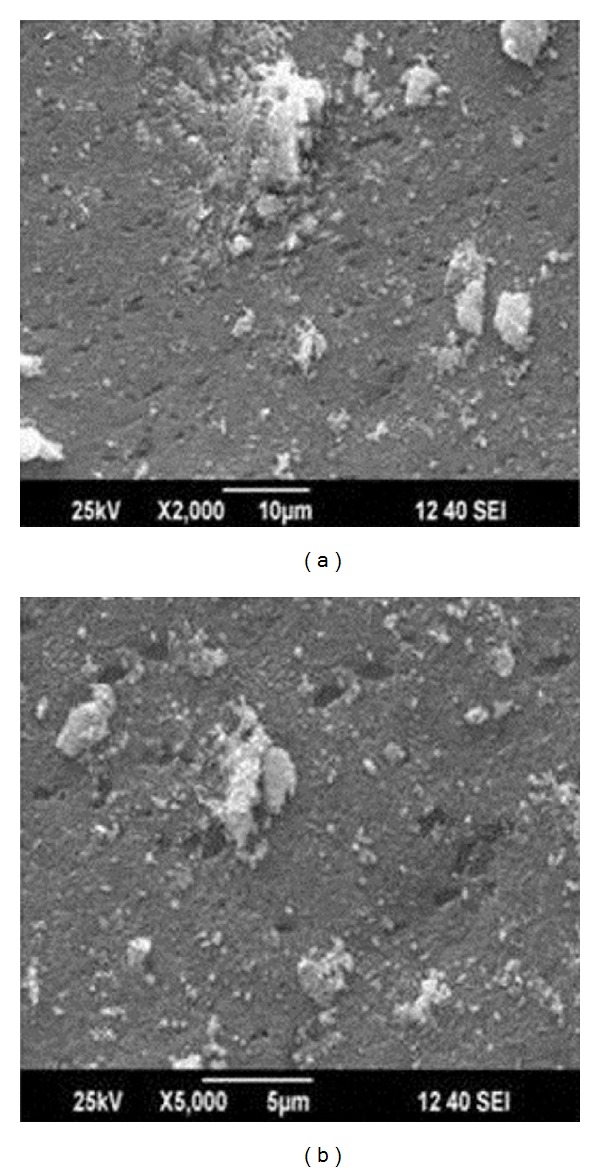
SEM image of silver nanoparticles shows spherical shape with agglomeration at different magnification, (a) 2,000x and (b) 5,000x.

Figures 4(a) and 4(b) show scanning electron microscope images of silver nanoparticles. The silver nanoparticles were viewed at different magnifications like 2,000x and 5,000x and the particle was approximately in the range of 1 nm to 10 nm. The morphology of silver nanoparticles is almost spherical in shape. The SEM image clearly indicates the particles were agglomerated and they formed irregular shape. Few particles were spherical like in structure. SEM image also shows the lot of agglomeration was found to probably be due to due to the presence of biomolecules of the culture supernatant from* Planomicrobium* sp. The SEM images of silver nanoparticles are spherical in shape. The clear structure was not found in the SEM image because the biomolecules responsible for the nanoparticles synthesis was found in the surface of the nanoparticles [20]. The same shaped nanoparticles were observed from Ag bimetallic nanoparticles synthesized from filamentous fungus* Neurospora crassa* [21].

### 3.5. EDX

**Figure 5:**
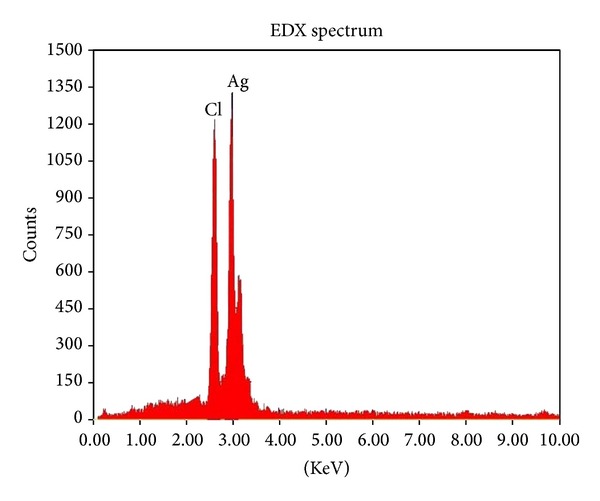
EDX analysis of silver nanoparticles.

Analysis through energy-dispersive X-ray analysis confirmed the presence of elemental silver. Strong signal at 3 KeV confirms the silver nanoparticles formation in the solution. The weak signals were observed for chloride due to the biochemical molecules of bacteria responsible for the silver nanoparticles synthesis (Figure 5).

### 3.6. TEM and SAED Analysis

**Figure 6:**
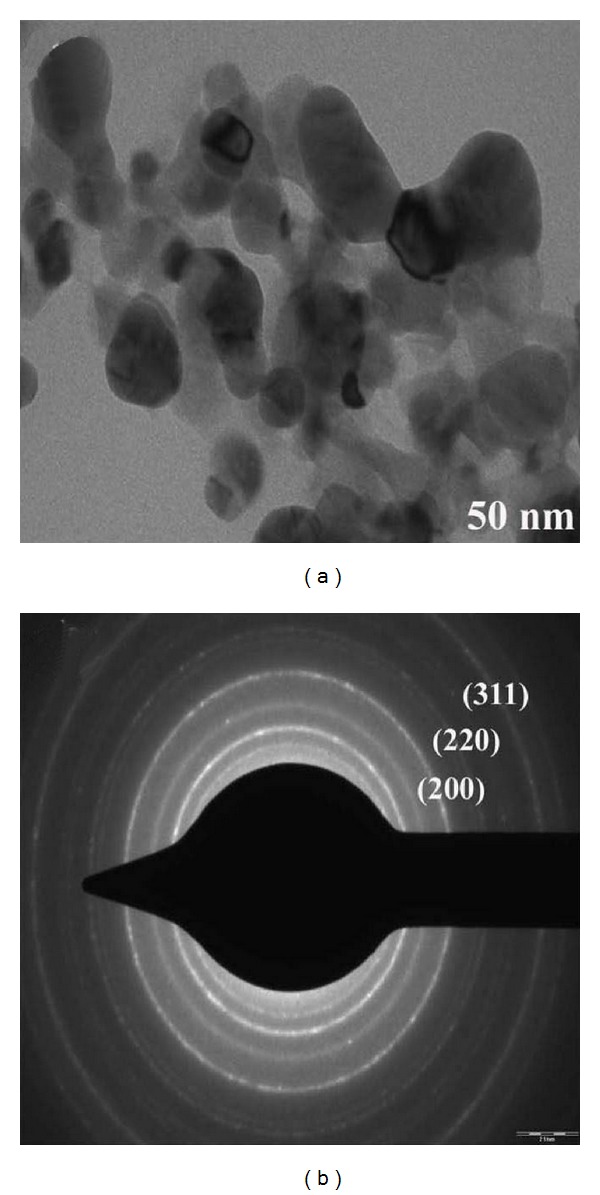
TEM image of silver nanoparticles shows spherical shape at 50 nm scale bar; (b) SAED pattern indicates crystalline nature of synthesized silver nanoparticles.

Morphological structure and distribution of synthesized silver nanoparticle were characterized at high magnifications done by TEM.  Figure 6(a) shows the well dispersed predominantly spherical shape of silver nanoparticles and also it confirms that the synthesized silver nanoparticles were in nanosize [22]. SAED pattern shows three rings corresponding to (200), (220), and (311) index of face centered cubic (FCC) structure in crystalline and these results are also reported by using XRD analysis (Figure 6(b)).

### 3.7. FTIR Analysis

**Figure 7:**
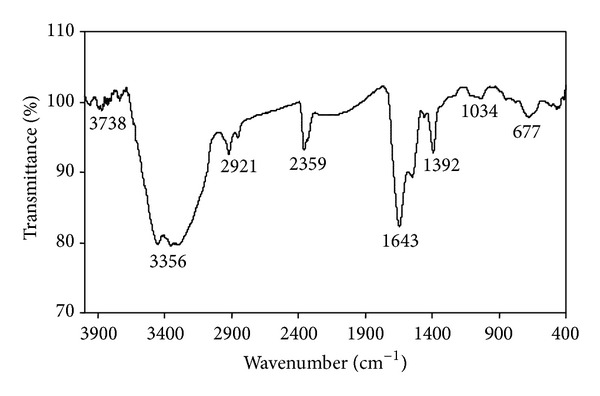
FTIR spectra of silver nanoparticle synthesized by* Planomicrobium *sp.

FTIR shows the biomolecules associated with silver nanoparticles which are responsible for reduction of silver ions to silver nanoparticles (Figure 7). A strong band which was observed at 3356 cm^−1^ represents the N–H stretching of amide linkages of proteins. Weak bands at 2921, 2351 cm^−1^ was observed due to the stretching vibrations of methylene groups and N–H stretching of amines, respectively. The narrow band at 1643 cm^−1^ corresponds to –C=C– stretching of alkenes. The small intense peak at 1392 cm^−1^ shows the presence of nitrocomponds from proteins or enzymes. The two weak bands are observed at 1034 and 677 cm^−1^ was assigned to aliphatic amines and –C=H bending of alkenes. FTIR spectrum confirms the presence of amide linkages of proteins, methylene groups of proteins or enzymes, and nitro compounds [8] which are functional biomolecules of proteins or enzymes from culture supernatant of* Planomicrobium *sp. Hence it was concluded that these functional molecules resulted from nitrate reductase enzyme may involve in the reduction of silver ions to silver nanoparticles. Similarly [17] explained and used purified nitrate reductase from* Fusarium oxysporum* for silver nanoparticles synthesis. Likewise, [23] also suggested that biomolecules components nitrate reductase enzyme is involved in reduction process and rhamnolipids is involved in capping the silver nanoparticles which are found in culture supernatant* Pseudomonas aeruginosa*.

### 3.8. Antibacterial Activity by Well Diffusion Method

**Figure 8:**
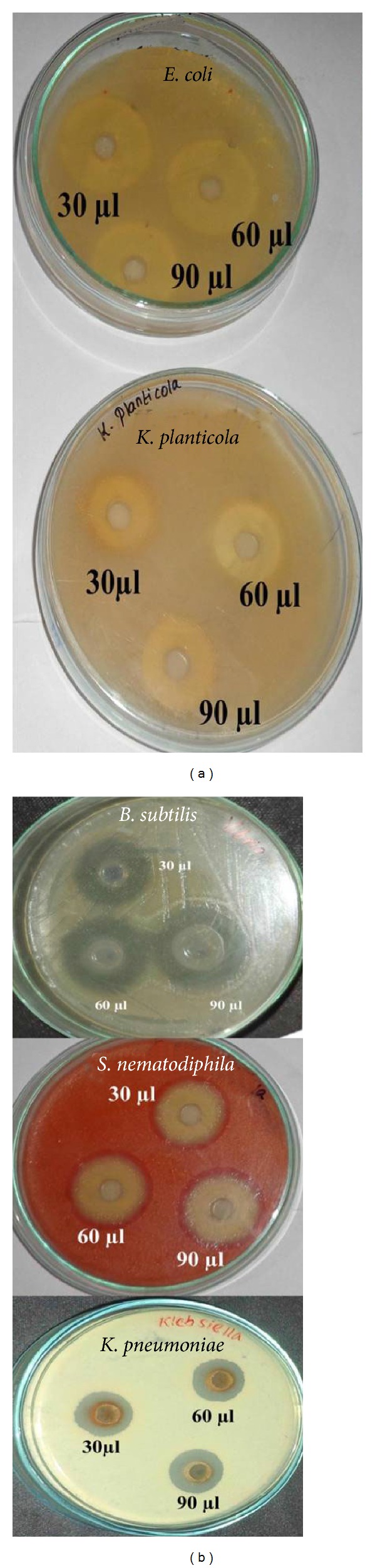
Antibacterial activity of silver nanoparticles against pathogens by agar well diffusion method.

Silver nanoparticles which were synthesized from marine bacterial strain having good zone of inhibitions were shown in Table 1 and Figure 8. The antibacterial activity conducted against the pathogenic bacteria such as* B. subtilis, K. planticola, K. pneumonia, S. nematodiphila, *and* E. coli*. There are three different concentrations (30 *μ*L, 60 *μ*L, and 90 *μ*L) which were taken to kill the pathogenic bacteria. In that bacterial synthesized AgNPs were energetically involved in the antibacterial activity against* B. subtilis, E. coli* and* S. nematodiphila* had the minimum zone of inhibition because of the maximum resistant capacity of the bacterial isolates. The medium range of inhibition was observed against* K. planticola *and* K. pneumonia* having lowest inhibition with the bacterial mediated silver nanoparticles. Silver is a metal known for its wide-ranging antimicrobial activity against gram positive and gram negative bacteria, including antibiotic resistant strains [24, 25]. It can be used to reduce infections in the action of burned areas [26–28], to bring to an end bacterium on food packaging, medical devices, and textile fabrics and for water treatment [29–32]. Silver nanoparticles, as an antibacterial agent, have been effective in a range of equipment, including glass, polymers, and titanium.

### 3.9. Antibacterial Effect Silver Nanoparticles Determined by Broth Dilution Method

**Figure 9:**
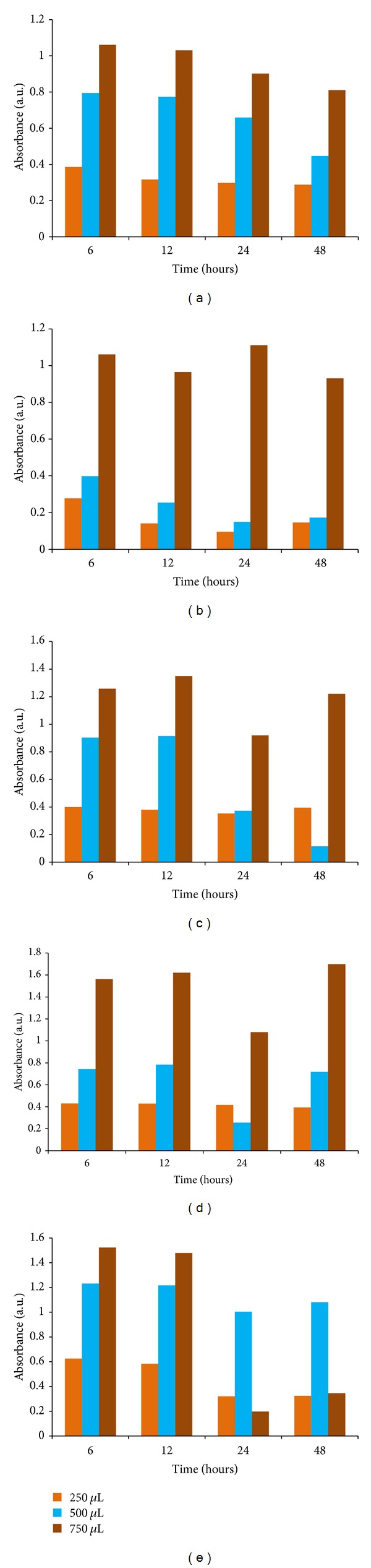
Antibacterial activity of silver nanoparticles by broth dilution method: (a)* B. subtilis,* (b)* K. planticola*, (c)* K. pneumoniae,* (d)* S. nematodiphila,* and (e)* E. coli*.

The antibacterial effect of silver nanoparticles against* B. subtilis, K. planticola, K. pneumoniae, S. nematodiphila, *and* E. coli* in LB broth was studied. The inhibitory growth effect of various concentrations of silver nanoparticles (250 *μ*L, 500 *μ*L, and 750 *μ*L) was examined through UV-vis spectrophotometer by taking optical density values (OD). The growth rate of* B. subtilis, K. planticola, K. pneumoniae, S. nematodiphila *and* E. coli* was decreased at the increased concentration of silver nanoparticles and the maximum inhibition of growth was obtained at 750 *µ*L (Figures 9(a), 9(b), 9(c), 9(d), and 9(e)). Antimicrobial activities that surround silver stand for a great challenge for academics and food industry. These materials novelty in life form a continuing antibacterial material with high temperature stability and low volatility [33]. The large enhancement in the amount and incident of antibiotic-resistant pathogen strains has motivated a renewed interest in the use of silver nanoparticles as an antibacterial agent [34].

**Figure 10:**
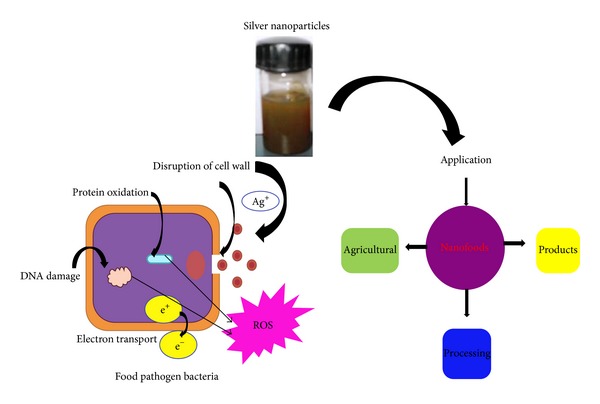
Mechanism of antibacterial activity of SNPs against food borne pathogens.

The bactericidal activity of nanoparticles can be related to several mechanisms (Figure 10). The silver nanoparticles may also directly interact with the microbial cells. Silver ions can be studied to uncouple respiratory electron transport from oxidative phosphorylation, which inhibits respiratory chain enzymes or interferes through covering permeability to phosphate and protons (e.g., interrupting transmembrane electron transfer, oxidizing cell components, disrupting, penetrating the cell covering or reactive oxygen species (ROS), or dissolving heavy metal ions that cause damage). The food industry is the major party concerned through the presence of pathogen strains, where crash to detect a pathogen may be directed to an awful effect. Even though the security of food has considerably enhanced overall, development of uneven and foodborne outbreaks from bacterial contaminations. However, many scientists and industry stakeholders' have already used silver nanoparticles in virtually every segment of the food industry from agriculture, processing, and products. This is possibly connected to the fact that the public has been shown in current studies to be more eager to embrace nanotechnology in “out of food” applications than those where silver nanoparticles are directly added to foods.

The present report showed a rapid and cost-effectiveness method to synthesize silver nanoparticles from* Planomicrobium* sp. The zone of inhibition clearly shows that the pathogenic strains tested are responsible for silver nanoparticles. The current report proved that the biologically silver nanoparticles seem to present potential and effective bactericidal covering material. This can be utilized in the direction of expanding the out crop life of food materials.

## 4. Conclusion

In conclusion, while the use of silver nanoparticles as a tool to detect the occurrence of contaminants or pathogen strains food protection is still in its infant, the present results are very promising in conditions of both detection limits. The outside of a normal packing material such as vegetables, paper, and plastic can be modified to make it suitable for food by surface coating it with many layers of tens to thousands of nanometers thickness. The present work have discussed many promising applications, including food packaging materials, antimicrobial properties, chemical contaminants, and more effective pesticides, and nanoencapsulate the release of nutrients and flavors. Further studies are needed for the commercialization.

## Figures and Tables

**Table 1:** Evaluation of antibacterial effects of silver nanoparticles against pathogenic bacteria.

S. number	Pathogenic bacteria	Concentrations of silver nanoparticles	Zone of inhibition (mm)
1	*B. subtilis *	30 *μ*L	17
		60 *μ*L	19
		90 *μ*L	21

2	*K. planticola *	30 *μ*L	14
		60 *μ*L	22
		90 *μ*L	23

3	*K. pneumoniae *	30 *μ*L	15
		60 *μ*L	18
		90 *μ*L	21

4	*S. nematodiphila *	30 *μ*L	21
		60 *μ*L	25
		90 *μ*L	29

5	*E. coli *	30 *μ*L	21
		60 *μ*L	23
		90 *μ*L	29
